# Supplementary material for: Salt-Induced Early Changes in Photosynthesis Activity Caused by Root-to-Shoot Signaling in Potato
Source: Int J Mol Sci. 2024 Jan 19;25(2):1229. doi: 10.3390/ijms25021229 (PMC10816847; doi:10.3390/ijms25021229)
Supplement: Supplementary file 1 [file ijms-25-01229-s001.zip › Figure S1.pdf]

Supplementary Material

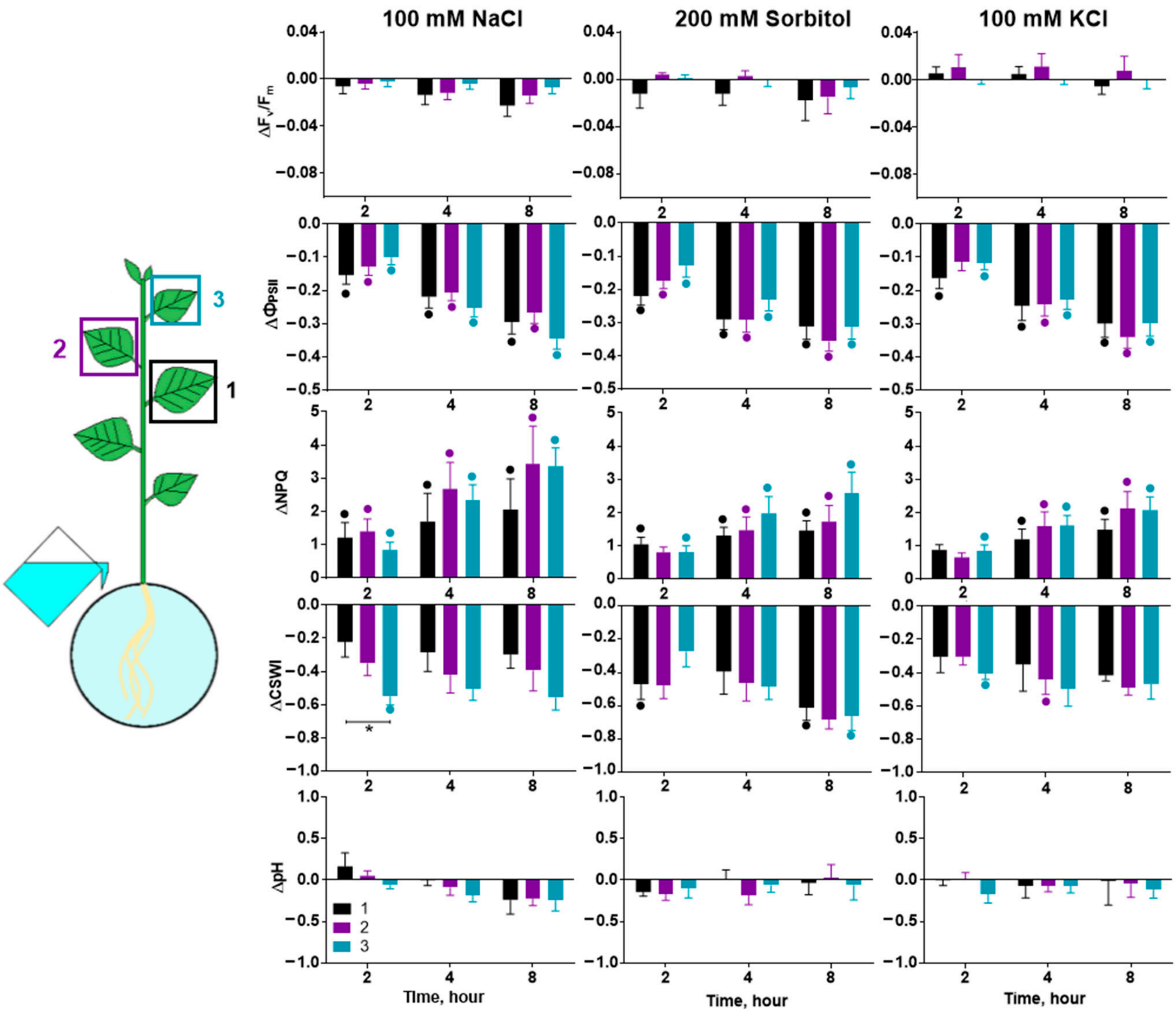

Figure S1. Effect of 100 mM NaCl, 200 mM sorbitol and 100 mM KCl on photosynthesis activity, transpiration and cytosolic pH in leaves of different strata (1, 2 and 3 in the scheme). Data represent differences in F_v/F_m, Φ_{PSII}, NPQ, CWSI or pH between treated and control plants. Data represent the mean ± SEM (*n* = 9), bullets (•) whose color corresponds to the column color indicate data significantly different (p<0.05) from the control, asterisk (*) indicates data significantly different (p<0.05) from the treatment.
