# Supplementary material for: Salt-Induced Early Changes in Photosynthesis Activity Caused by Root-to-Shoot Signaling in Potato
Source: Int J Mol Sci. 2024 Jan 19;25(2):1229. doi: 10.3390/ijms25021229 (PMC10816847; doi:10.3390/ijms25021229)
Supplement: Supplementary file 1 [file ijms-25-01229-s001.zip › Figure S10.pdf]

Supplementary Material

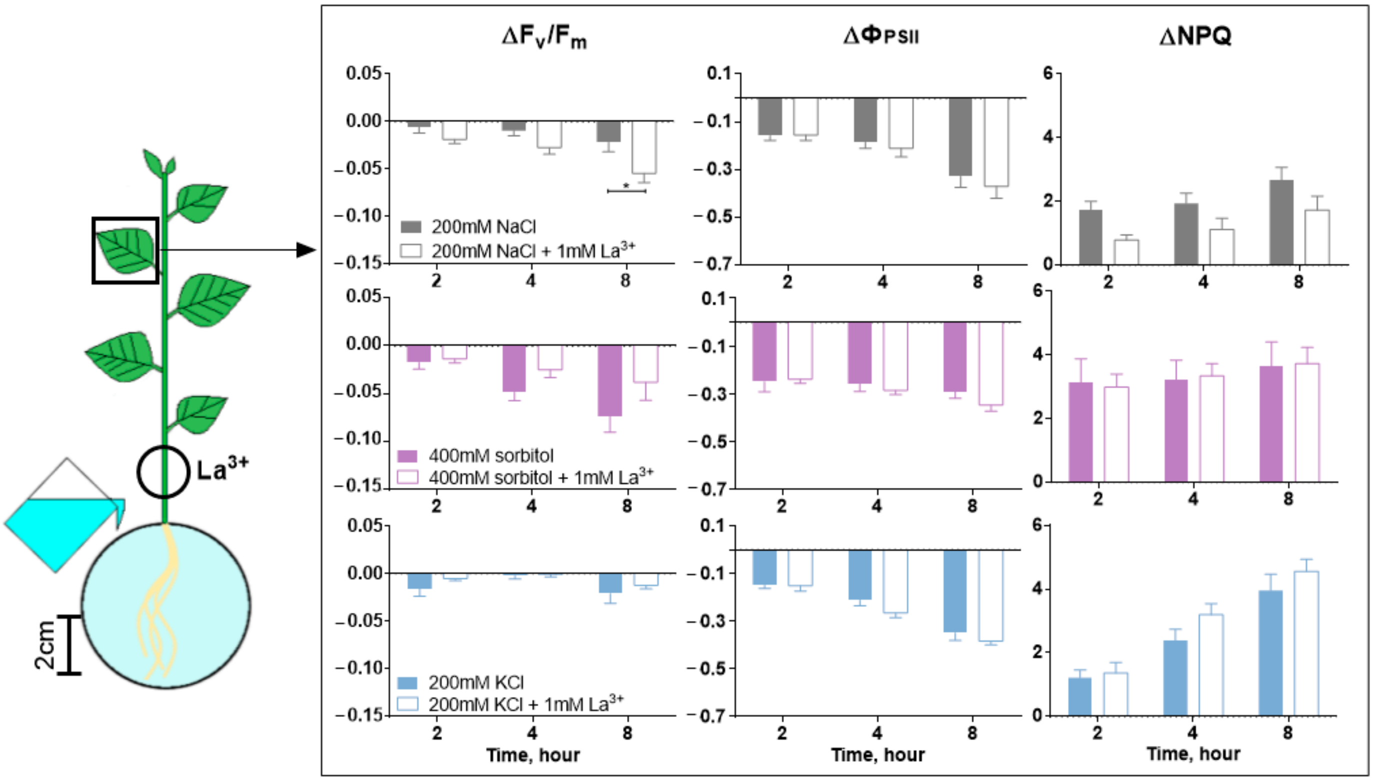

Figure S10. Influence 200 mM NaCl, 400 mM sorbitol or 200 mM KCl on photosynthesis activity in leaf with La^{3+} pretreatment and without. The circle shows a part of stem, which was incubated in La^{3+} solution. $\Delta F_v/F_m$, $\Delta\Phi_{PSII}$ or ΔNPQ represent the difference in F_v/F_m, Φ_{PSII} or NPQ between treated and control plants. Data represent the mean ± SEM (n = 9), * $p<0.05$ treatment versus treatment with La^{3+} pretreatment.
