# Supplementary material for: Salt-Induced Early Changes in Photosynthesis Activity Caused by Root-to-Shoot Signaling in Potato
Source: Int J Mol Sci. 2024 Jan 19;25(2):1229. doi: 10.3390/ijms25021229 (PMC10816847; doi:10.3390/ijms25021229)
Supplement: Supplementary file 1 [file ijms-25-01229-s001.zip › Figure S12.pdf]

Supplementary Material

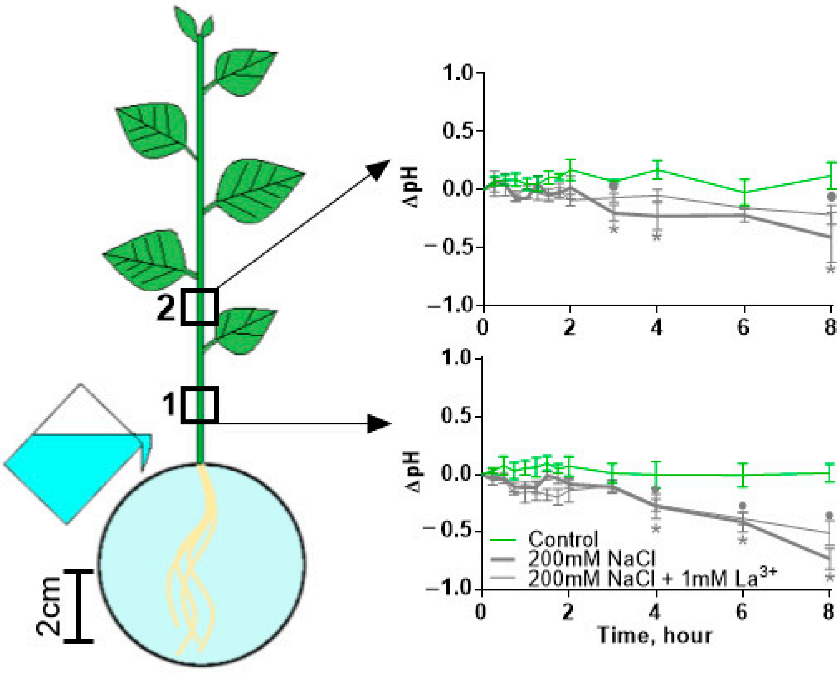

Figure S12. Changes in cytosolic pH induced by 200 mM NaCl in potato stem were determined in two part of stem (1 and 2). Cytosolic pH of plants treated by La^{3+} had similar dynamic. Control is plant treated with water. Data represent the difference in pH between time points before and after treatment. Data represent the mean ± SEM (n = 4). * $p < 0.05$ treatment versus control, • $p < 0.05$ treatment with La^{3+} pretreatment versus control.
