# Supplementary material for: Salt-Induced Early Changes in Photosynthesis Activity Caused by Root-to-Shoot Signaling in Potato
Source: Int J Mol Sci. 2024 Jan 19;25(2):1229. doi: 10.3390/ijms25021229 (PMC10816847; doi:10.3390/ijms25021229)
Supplement: Supplementary file 1 [file ijms-25-01229-s001.zip › Figure S13.pdf]

Supplementary Material

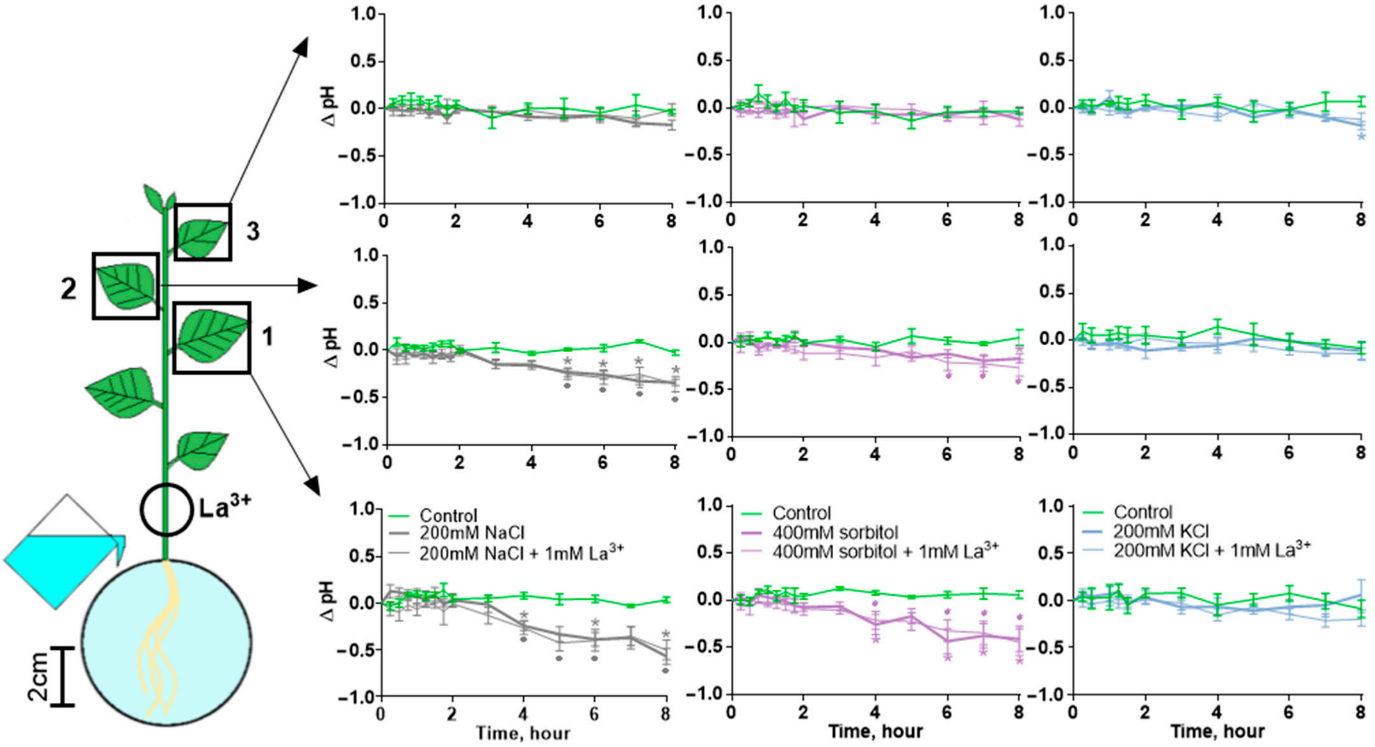

Figure S13. Changes in cytosolic pH induced by 200 mM NaCl, 400 mM sorbitol or 200 mM KCl in potato leaves. Cytosolic pH of plants treated by La^{3+} had similar dynamics. Control is plant treated with water. Data represent the difference in pH between time points before and after treatment. Data represent the mean ± SEM (n = 9), asterisks (*) whose color corresponds to the line color indicate data significantly different ($p < 0.05$) of treatment from the control, bullets (•) whose color corresponds to the line color indicate data significantly different ($p < 0.05$) of treatment with La^{3+} pretreatment from the control.
