# Supplementary material for: Salt-Induced Early Changes in Photosynthesis Activity Caused by Root-to-Shoot Signaling in Potato
Source: Int J Mol Sci. 2024 Jan 19;25(2):1229. doi: 10.3390/ijms25021229 (PMC10816847; doi:10.3390/ijms25021229)
Supplement: Supplementary file 1 [file ijms-25-01229-s001.zip › Figure S14.pdf]

Supplementary Material

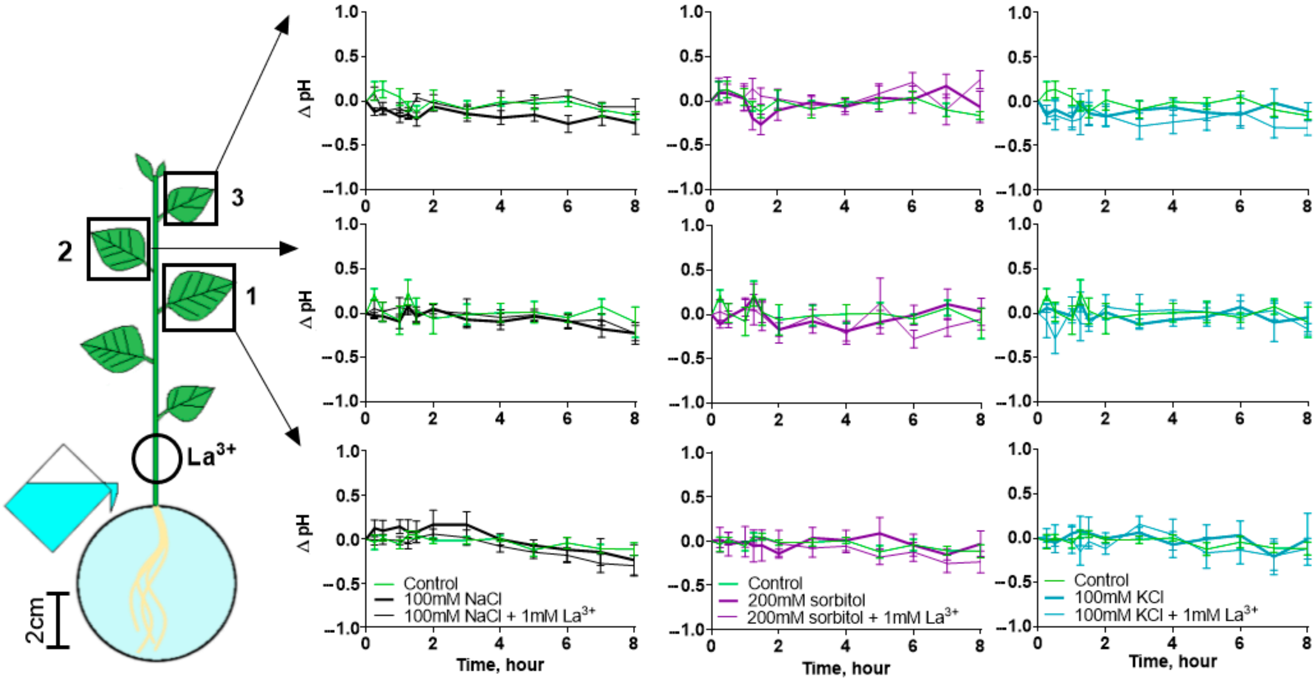

Figure S14. Changes in cytosolic pH induced by 100 mM NaCl, 200 mM sorbitol or 100 mM KCl in potato leaves. Cytosolic pH of plants treated by La^{3+} had similar dynamics. Control is plant treated with water. The circle shows a part of stem, which was incubated in La^{3+} solution. Data represent the difference in pH between time point before and after treatment. Data represent the mean ± SEM ($n = 9$).
