# Supplementary material for: Salt-Induced Early Changes in Photosynthesis Activity Caused by Root-to-Shoot Signaling in Potato
Source: Int J Mol Sci. 2024 Jan 19;25(2):1229. doi: 10.3390/ijms25021229 (PMC10816847; doi:10.3390/ijms25021229)
Supplement: Supplementary file 1 [file ijms-25-01229-s001.zip › Figure S15.pdf]

Supplementary Material

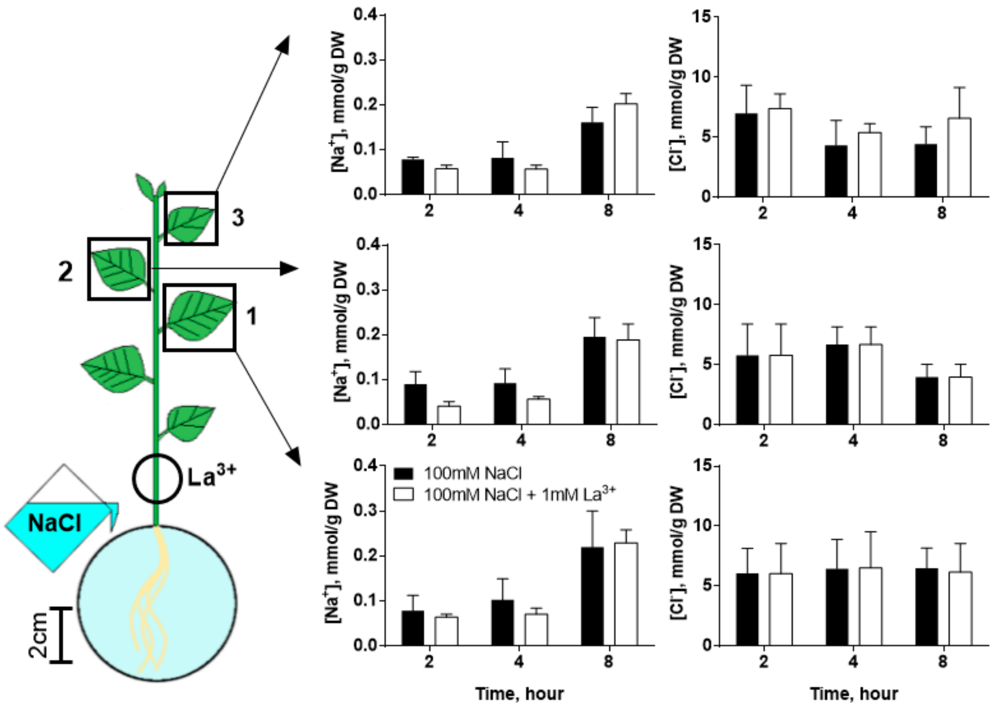

Figure S15. Accumulation of Na^+ and Cl^- in three leaves during 100 mM NaCl treatment in leaf with La^{3+} pretreatment and without. Data represent the mean ± SEM (*n* = 4, where *n* is biological replication, which included 5-7 leaves).
