# Supplementary material for: Salt-Induced Early Changes in Photosynthesis Activity Caused by Root-to-Shoot Signaling in Potato
Source: Int J Mol Sci. 2024 Jan 19;25(2):1229. doi: 10.3390/ijms25021229 (PMC10816847; doi:10.3390/ijms25021229)
Supplement: Supplementary file 1 [file ijms-25-01229-s001.zip › Figure S3.pdf]

Supplementary Material

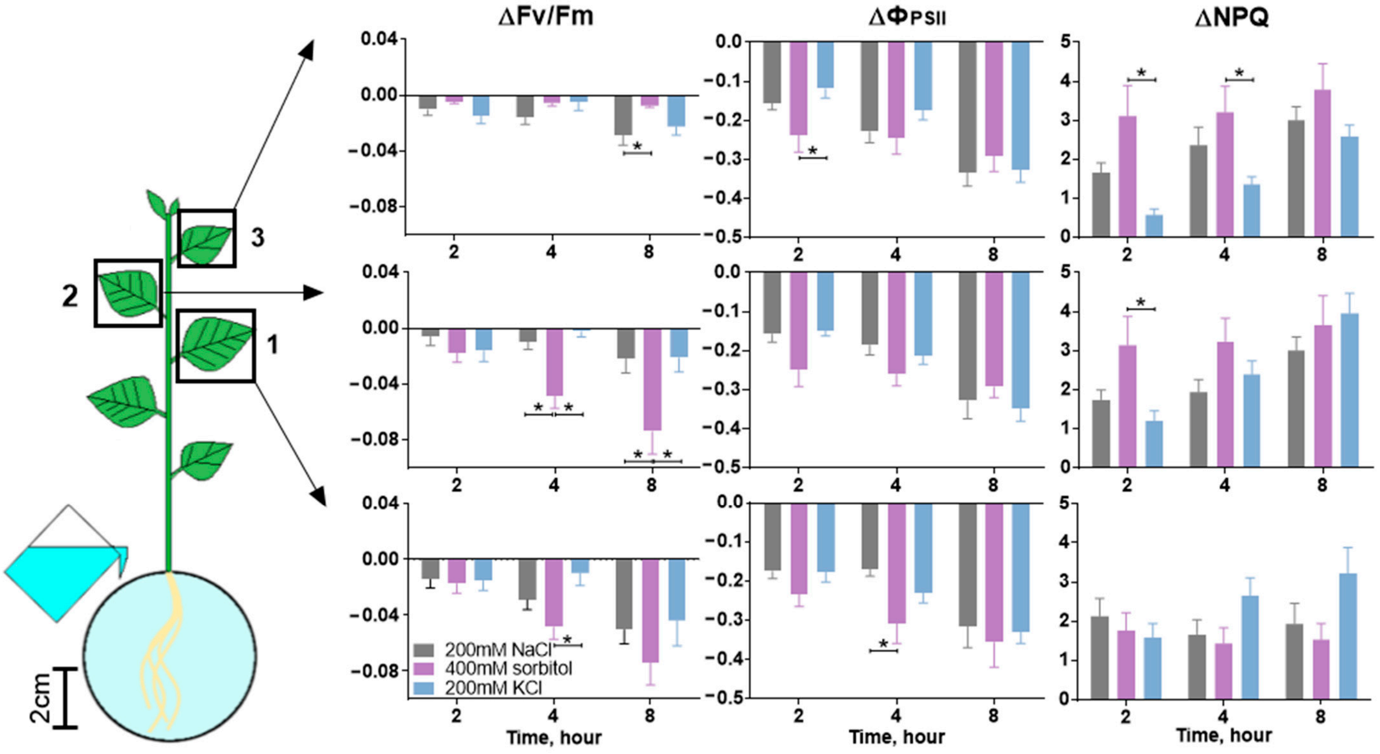

Figure S3. Comparison of 200 mM NaCl, 400 mM sorbitol and 200 mM KCl treatment in influence on photosynthesis activity. $\Delta F_v/F_m$, $\Delta\Phi_{PSII}$ or ΔNPQ represent differences in F_v/F_m, Φ_{PSII} or NPQ between treated and control plants. Data represent the mean ± SEM (n = 9). * $p < 0.05$ between the two parameters.
