# Supplementary material for: Salt-Induced Early Changes in Photosynthesis Activity Caused by Root-to-Shoot Signaling in Potato
Source: Int J Mol Sci. 2024 Jan 19;25(2):1229. doi: 10.3390/ijms25021229 (PMC10816847; doi:10.3390/ijms25021229)
Supplement: Supplementary file 1 [file ijms-25-01229-s001.zip › Figure S5.pdf]

Supplementary Material

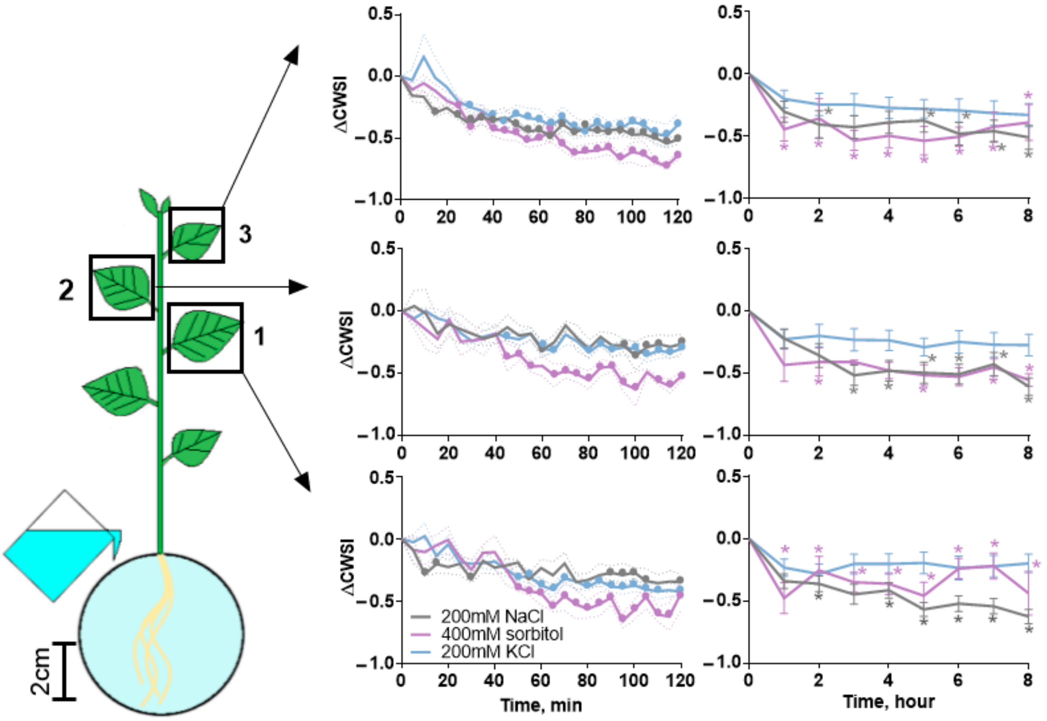

Figure S5. Changes in CWSI during 200 mM NaCl, 400 mM sorbitol or 200 mM KCl treatment in three potato leaves (1, 2 and 3 in the scheme). Data represent the difference in CWSI between time points before and after treatment. Data represent the mean ± SEM ($n = 6$), bullets (•) or asterisks (*) whose color corresponds to the line color indicate data significantly different ($p < 0.05$) from the control.
