# Supplementary material for: Salt-Induced Early Changes in Photosynthesis Activity Caused by Root-to-Shoot Signaling in Potato
Source: Int J Mol Sci. 2024 Jan 19;25(2):1229. doi: 10.3390/ijms25021229 (PMC10816847; doi:10.3390/ijms25021229)
Supplement: Supplementary file 1 [file ijms-25-01229-s001.zip › Figure S6.pdf]

Supplementary Material

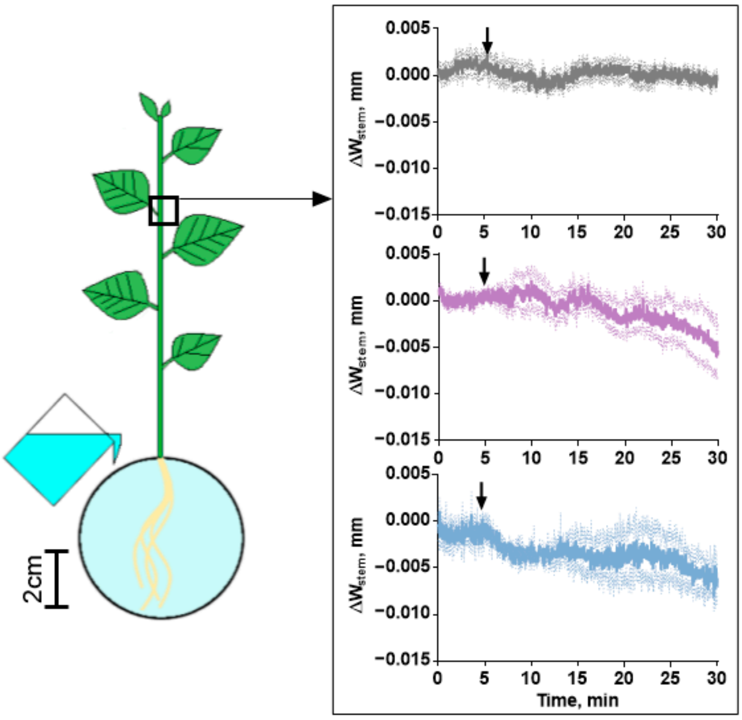

Figure S6. Changes in width of stem induced by 200 mM NaCl (gray), 400 mM sorbitol (gray purple) or 200 mM KCl (gray blue). The arrow indicates the moment of treatment. Data represent the mean ± SEM ($n = 5$).
