# Supplementary material for: Salt-Induced Early Changes in Photosynthesis Activity Caused by Root-to-Shoot Signaling in Potato
Source: Int J Mol Sci. 2024 Jan 19;25(2):1229. doi: 10.3390/ijms25021229 (PMC10816847; doi:10.3390/ijms25021229)
Supplement: Supplementary file 1 [file ijms-25-01229-s001.zip › Figure S7.pdf]

Supplementary Material

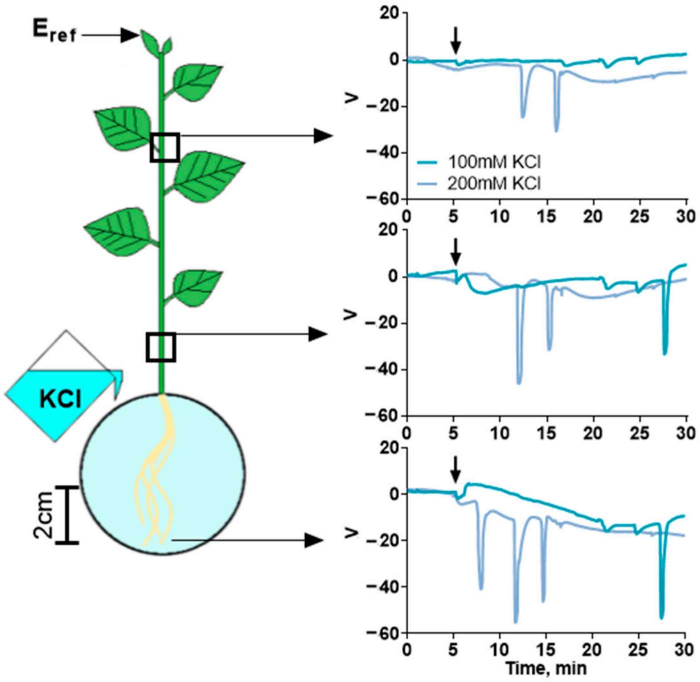

Figure S7. Changes in electrical potentials (V) induced by 100 mM KCl or 200 mM KCl in potato stem and roots. There are examples of generation of action potentials. Electrode of reference (E_{ref}) located by top leaf. The arrow indicates the moment of treatment.
