# Supplementary material for: Salt-Induced Early Changes in Photosynthesis Activity Caused by Root-to-Shoot Signaling in Potato
Source: Int J Mol Sci. 2024 Jan 19;25(2):1229. doi: 10.3390/ijms25021229 (PMC10816847; doi:10.3390/ijms25021229)
Supplement: Supplementary file 1 [file ijms-25-01229-s001.zip › Figure S8.pdf]

Supplementary Material

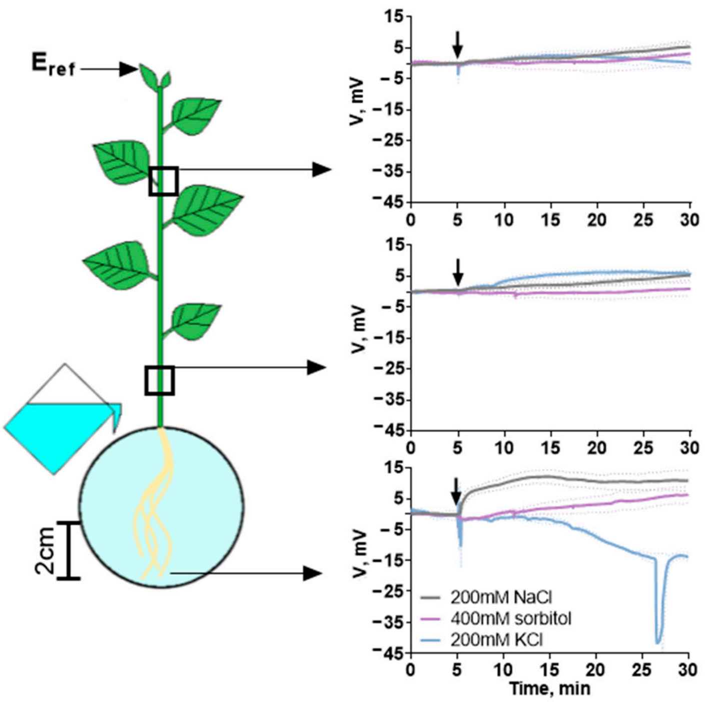

Figure S8. Changes in electrical potentials (V) induced by 200 mM NaCl, 400 mM sorbitol or 200 mM KCl in potato stem and roots. Reference electrode (E_{ref}) is located by top leaf. The arrow indicates the moment of treatment. Data represent the mean ± SEM (*n* = *5*).
