# Supplementary material for: Salt-Induced Early Changes in Photosynthesis Activity Caused by Root-to-Shoot Signaling in Potato
Source: Int J Mol Sci. 2024 Jan 19;25(2):1229. doi: 10.3390/ijms25021229 (PMC10816847; doi:10.3390/ijms25021229)
Supplement: Supplementary file 1 [file ijms-25-01229-s001.zip › Figure S9.pdf]

Supplementary Material

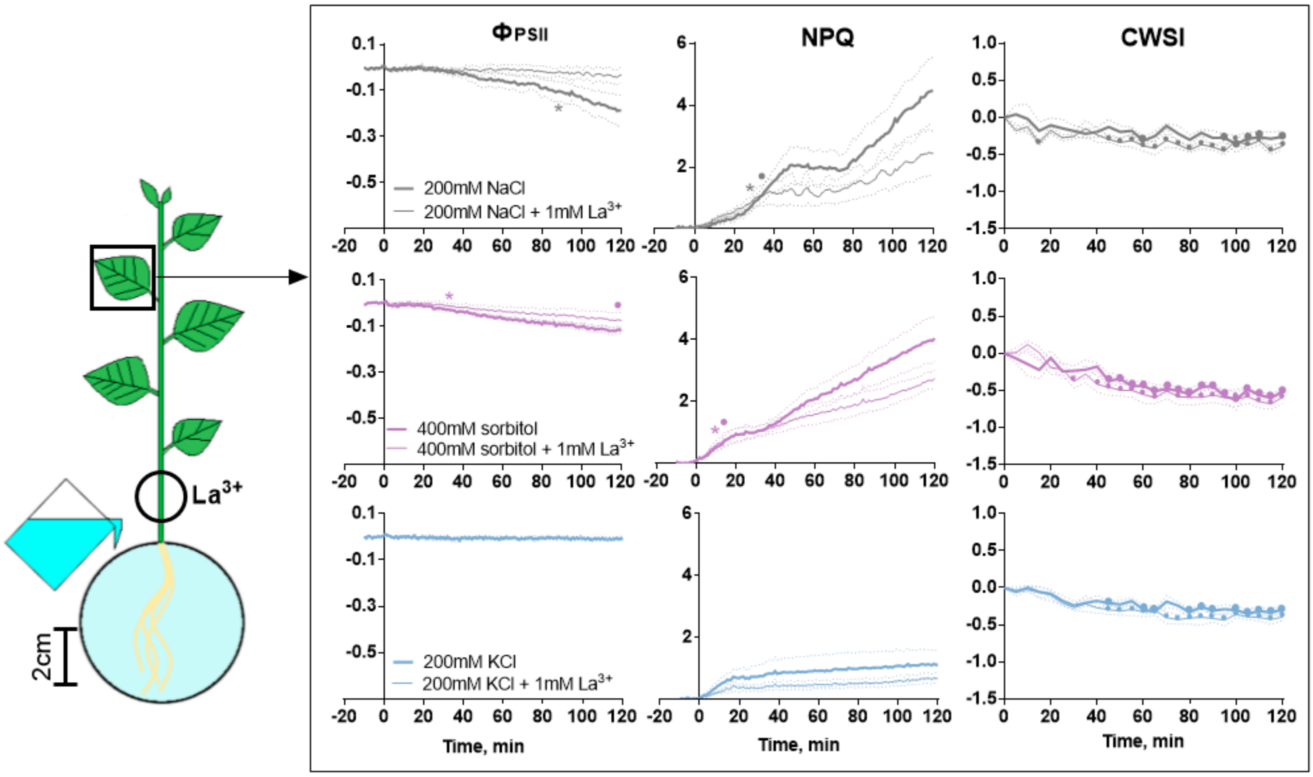

Figure S9. Influence of Ca^{2+} signaling inhibition on photosynthesis activity and transpiration during 200 mM NaCl, 400 mM sorbitol or 200 mM KCl treatment in leaf. Ca^{2+} signaling was inhibited by 1 mM La^{3+}. The circle shows a part of stem, which was incubated in La^{3+} solution. Data represent the mean ± SEM (n = 6), bullets (•) whose color corresponds to the line color indicate first data significantly different ($p < 0.05$) of treatment from the control, asterisks (*) whose color corresponds to the line color indicate first data significantly different ($p < 0.05$) of treatment with La^{3+} pretreatment from the control.
